# Academic and scientific education in medical curricula in the Netherlands: a programme director’s view

**DOI:** 10.1007/s40037-013-0076-x

**Published:** 2013-09-05

**Authors:** Arnout Jan de Beaufort, Anton F. P. M. de Goeij

**Affiliations:** 1 Leiden University Medical Center, V7-48, PO Box 9600, 2300 RC Leiden, the Netherlands; 2 Faculty of Health Medicine and Life Sciences, Maastricht University Medical Center, PO Box 616, 6200 MD Maastricht, the Netherlands

## Background

The role of scholar is one of the seven CanMEDS roles of the medical practitioner as described in the 2009 Framework for Undergraduate Medical Education in the Netherlands, adopted by all eight University Medical Centres (UMCs) [1]. In order to realize the desired competency level of ‘scholar’, academic and scientific training is an integral and substantial part of medical curricula [2].

We wish to share our reflections on the development of academic and scientific training in medical programmes, with emphasis on the undergraduate programme, triggered by rapidly expanding medical knowledge, and by the changing health care environment as described in the inspiring paper by Frenk et al. [3].

We address four basic questions regarding academic and scientific education: *what, why*, *when and how*.

## What

The 2009 Framework describes the scholarly abilities of future physicians in Chapter 5: ‘As scholars, physicians make scholarly contributions to the assessment, establishment, and understanding of knowledge and skills in health care. Physicians engage in teaching tasks and/or facilitate the education of their students, patients, and others. Whenever possible, physicians take clinical decisions based on a scientific footing, recognize the importance of life-long learning, and act as role models for others’ [1].

In addition, the Framework specifies that medical graduates are able to design and implement a small-scale empirical scientific research project, i.e. formulate a problem definition and a research question, conduct a literature survey, draft a simple methodologically sound research design, collect data, perform simple data cleaning and data entry tasks, perform simple statistical analysis, report research results in writing and present and discuss research outcomes.

The above abilities should be attained at the end of 6 years of medical education and serve as a solid basis for life-long learning. Academic and scientific training aims at development of a critical academic attitude and scientific skills of the medical student. This will lead to, at the very least, a critical user and applier of science in medical practice, and often to the development of a researcher who will ‘produce’ science.

## Why

Future physicians are expected to perform as academic professionals in medical practice using evidence-based medicine wherever possible.

From an educational viewpoint we consider the following qualities pivotal for our graduates:

- The ability of independent, critical thinking and reflection. This ability includes adequate problem analysis and solving, dealing with limitations of and fallacies in diagnostic results, the critical interpretation of medical literature and commercial information, and the awareness of lack of knowledge or skills and one’s own blind spots.
- The ambition to understand the nature of the patient’s complaints, symptoms and needs.
- The curiosity to ask questions and develop ideas helping to define and to design new research directives to improve care and prevention.
- The ability to actively participate in research, varying from basic projects to clinical studies.
- The ability to convey expertise to colleagues, students, patients and others, i.e. teaching qualities.

Students at university medical centres or medical schools are part of larger academic communities. Not all students will pursue academic careers later in life. However, as medical professionals, no matter in which practice their future will be, they are expected to have attained a firm academic and scientific basis during their education at medical school and thereafter. To this end their curriculum should include guidance during theoretical instruction in basic sciences, as well as in clinical sciences. They should have the opportunity to meet a variety of scientists for informal and critical discussions on scientific approaches, results and conclusions. Guided hands-on training focused on application of acquired academic and scientific competencies is an essential part of the educational programme.

Studying medicine is popular amongst talented students. These students are usually well-motivated and will flourish in a stimulating academic and scientific environment, supported by a programme which offers intellectual challenges and opportunities to participate in research projects, locally and internationally. Taken together, academic and scientific development is a sound basis for life-long learning.

Teachers play a crucial role in the academic and scientific development of students as guides and supporters. They make an important contribution to the realisation of the aforementioned student goals.

In the cycle of life-long learning, teachers have the privilege—and in our view also the obligation (1) to convey their research and teaching expertise to the next generation of health professionals and researchers, (2) to share their scientific abilities and enthusiasm for research and teaching and (3) to think beyond the obvious and stimulate curiosity.

The reciprocal is true as well: teacher-researchers will profit from students’ unconventional and creative thinking and should enjoy challenging and inspiring students to critically think and discuss interesting scientific problems. Moreover, teaching offers scientific investigators the opportunity of talent scouting and identifying future PhD students and colleagues in research projects. We think this is an essential asset for maintaining and further improving the quality of scientific research.

Patients want—and deserve—physicians who provide the best available care, physicians who continuously seek to better understand the nature of the patient’s condition and physicians who are able to critically reflect on their own abilities, both their strengths and their limitations. Patients expect -and deserve- best care at the individual level and, more indirectly, the identification and execution of preventive measures at the population level, the latter being equally relevant from the societal perspective.

The fundamental skills of critical thinking and understanding are crucial for every physician at every stage of professional life. Once properly seeded during the medical curriculum it is the challenge for each professional to keep these abilities active life-long. Each physician should at any time ask him or herself: ‘Do I (still) understand the nature of the patient’s problem?’

Physicians have an important role in society. Developments in research as well as a scientific approach in public health contribute significantly to the quality and effectiveness of medical care and preventive measures.

General problems in medical practice, such as patient safety and the need for the reduction of costs of the health care system, will require research to find appropriate solutions and medical leadership to address these issues.

## When and how

The medical curricula in the Netherlands have adopted a three-year Bachelor’s and three-year Master’s structure, in line with the Bologna agreement. The Bachelor-Master structure aims to facilitate movement of students and research is a good opportunity to cross borders, by intensifying existing and initiating new international exchange programmes for research and education.

In order to provide a thorough understanding of the human organism and its relation to the environment, Bachelor’s programmes provide integrated basic and clinical sciences courses, usually with the emphasis on the basic sciences. Basic scientists have teaching roles in many courses and convey scientific reasoning often implicitly. Students will profit when their academic and scientific abilities are explicitly challenged.

In most, if not all, Bachelor’s courses academic and scientific development can and should be an integral part of the course learning goals. Early seeding of academic and scientific abilities for all medical students will contribute to fruitful medical careers. Research electives as part of mandatory electives in Bachelor’s programmes offer great opportunities to engage in academic and scientific development.

During Dutch Master’s programmes all students dedicate on average 3 months (ranging from 14 to 24 weeks) to an individual research project of their choice. Students write a research proposal, which they carry out themselves, perform experiments and analyse and report the data. This enables them to develop their scientific skills ‘hands on’, by doing, presenting and writing guided by a scientist.

Both Bachelor’s and Master’s courses offer opportunities to develop and engage in teaching qualities, i.e. literature searches, EBM teaching, critical appraisal of a topic (‘CAT’), presentations on the basis of assignments and internships should be used extensively.

## Conclusion

We consider academic and scientific development to be a key element in medical education. Academic and scientific competencies are vital for proper care at the individual as well as the population level. Patients and society require curious, independent and critically thinking physicians with a scientific attitude and the ability to transfer expertise. To achieve this goal medical curricula should offer challenging scholarly programmes that match and stimulate academic and scientific talents of students and teachers.
